# What’s in a Name? Species-Wide Whole-Genome Sequencing Resolves Invasive and Noninvasive Lineages of *Salmonella enterica* Serotype Paratyphi B

**DOI:** 10.1128/mBio.00527-16

**Published:** 2016-08-23

**Authors:** Thomas R. Connor, Sian V. Owen, Gemma Langridge, Steve Connell, Satheesh Nair, Sandra Reuter, Timothy J. Dallman, Jukka Corander, Kristine C. Tabing, Simon Le Hello, Maria Fookes, Benoît Doublet, Zhemin Zhou, Theresa Feltwell, Matthew J. Ellington, Silvia Herrera, Matthew Gilmour, Axel Cloeckaert, Mark Achtman, Julian Parkhill, John Wain, Elizabeth De Pinna, François-Xavier Weill, Tansy Peters, Nick Thomson

**Affiliations:** a Cardiff University School of Biosciences, Cardiff University, Cardiff, United Kingdom; b Wellcome Trust Sanger Institute, Hinxton, United Kingdom; c Institute of Integrative Biology, University of Liverpool, Liverpool, United Kingdom; d Gastrointestinal Bacteria Reference Unit, Public Health England, London, United Kingdom; e Public Health England, London, United Kingdom; f Department of Mathematics and Statistics, University of Helsinki, Helsinki, Finland; g Bacteriology and Enteric Disease Program, National Microbiology Laboratory, Public Health Agency of Canada, Winnipeg, Canada; h Institut Pasteur, Unité des Bactéries Pathogènes Entériques, Paris, France; i INRA, UMR1282 Infectiologie et Santé Publique, Nouzilly, France; j Université François Rabelais de Tours, UMR1282 Infectiologie et Santé Publique, Tours, France; k Warwick Medical School, University of Warwick, Coventry, United Kingdom; l Public Health England, Addenbrooke’s Hospital, Cambridge, United Kingdom; m Instituto de Salud Carlos III, Centro Nacional de Microbiología, Majadahonda, Madrid, Spain; n Norwich Medical School, UEA, Norwich, United Kingdom; o London School of Hygiene and Tropical Medicine, London, United Kingdom; p Department of Biostatistics, University of Oslo, Oslo, Norway

## Abstract

For 100 years, it has been obvious that *Salmonella enterica* strains sharing the serotype with the formula 1,4,[5],12:b:1,2—now known as Paratyphi B—can cause diseases ranging from serious systemic infections to self-limiting gastroenteritis. Despite considerable predicted diversity between strains carrying the common Paratyphi B serotype, there remain few methods that subdivide the group into groups that are congruent with their disease phenotypes. Paratyphi B therefore represents one of the canonical examples in *Salmonella* where serotyping combined with classical microbiological tests fails to provide clinically informative information. Here, we use genomics to provide the first high-resolution view of this serotype, placing it into a wider genomic context of the *Salmonella enterica* species. These analyses reveal why it has been impossible to subdivide this serotype based upon phenotypic and limited molecular approaches. By examining the genomic data in detail, we are able to identify common features that correlate with strains of clinical importance. The results presented here provide new diagnostic targets, as well as posing important new questions about the basis for the invasive disease phenotype observed in a subset of strains.

## IMPORTANCE

*Salmonella enterica* strains carrying the serotype Paratyphi B have long been known to possess Jekyll and Hyde characteristics; some cause gastroenteritis, while others cause serious invasive disease. Understanding what makes up the population of strains carrying this serotype, as well as the source of their invasive disease, is a 100-year-old puzzle that we address here using genomics. Our analysis provides the first high-resolution view of this serotype, placing strains carrying serotype Paratyphi B into the wider genomic context of the *Salmonella enterica* species. This work reveals a history of disease dating back to the middle ages, caused by a group of distinct lineages with various abilities to cause invasive disease. By quantifying the key genomic differences between the invasive and noninvasive populations, we are able to identify key virulence-related targets that can form the basis of simple, rapid, point-of-care tests.

## INTRODUCTION

*Salmonella paratyphosus* B, *Salmonella schottmuelleri*, *Salmonella* Java: the serotype of *Salmonella enterica* subspecies *enterica* with the formula 1,4,[5],12:b:1,2 that is now known as *S. enterica* serotype Paratyphi B has not always been named thus. In the last 100 years, it has been known by various names, several of which are still commonly used today. The reason for this multiplicity of names is because isolates possessing the serotype have long been observed to cause either invasive disease (characterized by life-threatening paratyphoid fever) or gastroenteritis. It was clear to many microbiologists in the late 19th and early 20th centuries that, despite the shared serotype, there were differences between strains that related to the different disease outcomes. However, categorizing the differences in the form of classical, reproducible biochemical tests has proven to be a nontrivial problem, as the differences are more subtle than the disease phenotypes might suggest. Muller and, later, Kauffmann ultimately subdivided the serotype into two biovars on the basis of an ability to form a slime wall and to ferment dextrorotatory tartrate (*d*Ta) (1, 2). In his classification of *Salmonella* isolates, Kauffmann named those isolates that formed a slime wall, were unable to ferment *d*Ta (*d*Ta^−^), and caused paratyphoid fever in humans *S*. Paratyphi B. Those isolates that did not form a slime wall and were able to ferment *d*Ta (*d*Ta^+^), he named *S*. Java (3). The already unclear delineation was further confused when Le Minor (4) ultimately rejected this nomenclature to redefine *S*. Java as a biovar of *S*. Paratyphi B. In practice, the result of the *d*-tartrate test remains the principal method for distinguishing *S*. Paratyphi B isolates causing invasive disease from those causing gastroenteritis (5). The implications of an isolate being classified as *S*. Paratyphi B or *S*. Java are significant for patients, reference laboratories, and public health authorities. Although in the first instance, treatment will depend on presentation, as a member of the so-called typhoidal *Salmonella* group, cases of disease where *S*. Paratyphi B is detected generally necessitate household follow-up and contact tracing. This is not required for *S.* Java infections, even when these cause systemic infections. In laboratory research, there are also significantly different handling requirements depending on the result—*d*Ta^−^ strains are treated as biological safety level 3 (BSL3) organisms, while *d*Ta^+^ organisms are handled at BSL2.

The molecular explanation for the differences in the ability to ferment *d*-tartrate is known: one single-nucleotide polymorphism (SNP) in the first codon of the gene upstream from *ttdA* and *ttdB*, the genes responsible for *d*-tartrate metabolism, ablates their expression (5). Based on IS*200* profiling (6), multilocus sequence typing (MLST) (7), and latterly, clustered regularly interspaced short palindromic repeat (CRISPR) typing (8), it is clear that the serotype falls into a number of discrete groups (7) and that possession of the *d*Ta^−^ SNP is characteristic of groups of strains that are predominantly associated with invasive disease. However, the relationship between groups of isolates carrying the common serotype remains unresolved (9). 

*Salmonella* strains possessing this serotype remain a common cause of gastroenteritis, being responsible for recent outbreaks in the United Kingdom (10), Belgium (11), Scandinavia (12), Canada (13, 14), and the United States (15), as well as a cause of invasive disease around the world in travelers (16). Moreover, since the late 1990s, two different clones of *S*. Paratyphi B *d*Ta^+^ with resistance to multiple antibiotics have become increasingly associated with human infections (17), poultry, and poultry products (11, 18). These clones carry two different multidrug resistance-encoding integrons, *Salmonella* genomic island 1 (SGI-1) (19) and a chromosomally located class 2 integron carrying the *dfrA1*-*sat2*-*aadA1* (Tn*7*) array of gene cassettes (20), which confer resistance to trimethoprim, streptothricin, and aminoglycosides, respectively. Because of an inability to unambiguously subdivide this grouping in a phylogenetically meaningful way and an almost complete lack of knowledge about the genomic content and phylogenetic relationships of these strains, the significance of these observations is difficult to quantify. The work presented here defines the population structure of strains carrying the Paratyphi B serotype and reveals how strains carrying this serotype can be subdivided and how they vary in gene content. By gaining a better understanding of the population structure of strains carrying this Paratyphi B serotype, we are able to define what separates invasive from noninvasive strains, opening up new opportunities to better diagnose and track this organism.

## RESULTS AND DISCUSSION

### Whole-genome sequencing reveals the extent of divergence between isolates sharing the Paratyphi B serotype.

**FIG 1:**
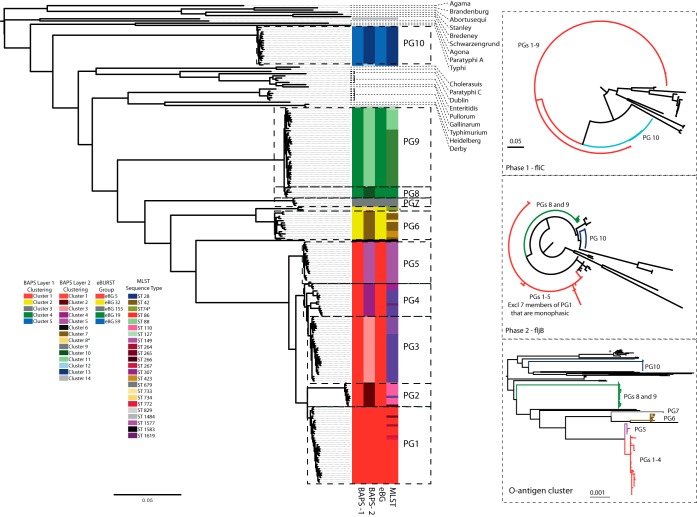
Whole-genome and LPS gene maximum-likelihood phylogenies of strains carrying serotype Paratyphi B and reference strains from other serotypes. Main figure: whole-genome phylogeny of the Paratyphi B strains, with representatives from other serogroup B and invasive serotypes shown. The tree was constructed based on the core genome for the isolates and drawn using RAxML. Next to the tree, colored blocks indicate the BAPS cluster that an isolate has been assigned to and the MLST and eBURST group that an isolate belongs to. Right, top to bottom: maximum-likelihood trees based on the nucleotide sequences of the phase 1 antigen *fliC* and the nucleotide sequences of *fljB*, drawn using PhyML, and maximum-likelihood tree based on the nucleotide sequences for the O-antigen cluster extracted from Paratyphi B and reference strains used to generate the whole-genome phylogeny, drawn using RAxML. In all cases, the phylogenetic trees were generated using a general time-reversible (GTR) model with gamma correction for between-site heterogeneity.

Based upon its O antigen (formula 1,4,[5],12), *S*. Paratyphi B is a member of the group O:4 (formally group B) salmonellae. Forty-six different O serogroups have been identified within *Salmonella* (21), and these serogroups provide a structure to group together the >2,500 serotypes of the species (22). To quantify the population structure of *S*. Paratyphi B, we assembled a collection of 191 strains collected over 120 years that possess the serotype with the formula 1,4,[5],12:b:[1,2], encompassing both diphasic (b:1,2-type flagella present) and monophasic (b-type flagella only) Paratyphi B/Java isolates. To place these samples into a wider context, we also included a selection of 25 other salmonellae, including 10 other representatives of group O:4, as well as published reference genomes for 6 other *Salmonella* serotypes associated with invasive disease in humans and animals (see Table S1 in the supplemental material). We began our analysis by identifying the core and accessory genomes across the isolate collection. The diversity of isolates carrying this serotype is evidenced in the fact that the pangenome of the serotype itself is open (see Fig. S1) (23) and the core genome size of isolates sharing the Paratyphi B serotype (2,949 genes) is smaller by almost 1,000 genes than those reported for other serotypes, such as *S. enterica* serotype Typhimurium (3,846 genes) (24). Relative to the core genome across the sample data set, approximately 53% of the genome of the *S*. Paratyphi B reference strain is core to the serotype, in comparison to 43% that is core to the entire sample data set. Following the removal of putative recombinant regions using Gubbins (see Fig. S1) (25), a phylogenetic analysis based on the remaining SNPs found at positions that are shared by all isolates visualizes the extreme level of diversity between isolates carrying the Paratyphi B serotype (Fig. 1). However, it is also clear that there is not a continuum of diversity present within this group, but rather, strains carrying the common serotype fall into discrete clusters of strains. Recognizing that classical subdivisions have introduced a confused and inconsistent nomenclature to describe this group, we used a population genetic statistical framework, Bayesian Analysis of Population Structure (BAPS) (26), to perform an unsupervised subdivision of the Paratyphi B complex into a set of phylogroups (PGs). Iteratively clustering across the population, BAPS identified a set of 10 Paratyphi B clusters that we define as PG1 to PG10. Only isolates from PG1 possessed the SNP that is diagnostic for *d*Ta^−^ strains. This represents the first occasion, to our knowledge, where an automated approach has unambiguously separated out *d*Ta^−^ strains into a cluster that is distinct from other closely related strains without the need for a marker such as the *d*Ta^−^ SNP to be provided as a basis for subdivision. Our results show that the *d*Ta^−^ isolates fall within a group of 5 PGs (PG1 to -5) that, although separated by between 1,000 and 10,000 SNPs, are more closely related to one another than they are to other PGs or serotypes. Intriguingly, this difference is of a scale similar to the ~6,000 SNPs that separate the closely related host generalist and invasive serotypes *S. enterica* serotypes Enteritidis and Gallinarum/Pullorum (see Fig. S2) (27). Moving beyond PG1 to -5, it is clear that the serotype 1,4,[5],12:b:[1,2] is found in genomic backgrounds across the *S. enterica* species tree (Fig. 1) and that all isolates sharing this serotype clearly do not share a recent common ancestor. The extent of the separation between PGs is marked: isolates from *S. enterica* serotypes Heidelberg, Derby, Paratyphi C, Choleraesuis, Gallinarum var. Gallinarum, Gallinarum var. Pullorum, Enteritidis, and Dublin all share more SNPs with PG1 to -9 than the isolates of PG10 do.

### The common serotype is a result of recombination events at the flagellum loci.

Kauffmann suggested that the evident variability in the genetic background of Paratyphi B strains may be due to recombination (28). The Paratyphi B serotype is defined based on its O antigen (responsible for the first part of the serotype formula, 4,5,12) and phase 1 and phase 2 flagella (the H antigens). The phase 1 flagella are of type b, and either the phase 2 flagella are of type 1,2 or, in the case of monophasic isolates, no phase 2 is present. Extracting the complete O-antigen gene cluster, as well as the *fliC* (phase 1) and *fljB* (phase 2) genes, and generating a phylogeny of these components individually revealed a startling similarity in the topology of the O-antigen cluster compared with the core genome tree (Fig. 1, inset). In contrast, the phylogenies for *fliC* (Fig. 1, inset) and *fljB* (Fig. 1, inset) individually revealed evolutionary histories that were markedly different from those of both the core genome and the O-antigen gene cluster. In the case of *fljB*, nonmonophasic Paratyphi B isolates all possessed identical or nearly identical gene sequences (Fig. 1, inset). Looking at the 20 kb of DNA around these two genes reveals a topology that is markedly different from the core genome tree (see Fig. S3 in the supplemental material), implying that while the common flagella with limited diversity could be due to selection, the more parsimonious explanation for the lack of diversity within *fljB* and *fliC* is that of homologous recombination. These results suggest that, in a number of cases, the import of flagellum genes has led to the establishment of clear lineages that have been able to cause widespread disease in humans and animals. It is, however, also interesting to note that when collecting isolates sharing the serotype 1,4,[5],12:b:[1,2] from clinical settings, we also collected a small number of strains that are singletons (Fig. 1, highlighted in grey). Of these cases, one strain appears to be novel, with an MLST profile that was uncharacterized previously. We observe that this strain is most closely related (1,191 SNPs; see Fig. S2) to a sample that has been serotyped as *S*. Derby and so may represent a serotype switch from *S*. Derby (antigenic formula 1,4,[5],12:f,g:[1,2]), based on the acquisition of the b-type phase 1 flagella. In the case of the other singleton, no close relatives were identified, although other isolates sharing the same sequence type are recorded in the MLST database with a Paratyphi B serotype, suggesting that this observation is not simply the result of a sequencing or serotyping error.

### *d*Ta^−^ strains have limited differences in their virulence repertoire compared with close relatives.

**FIG 2:**
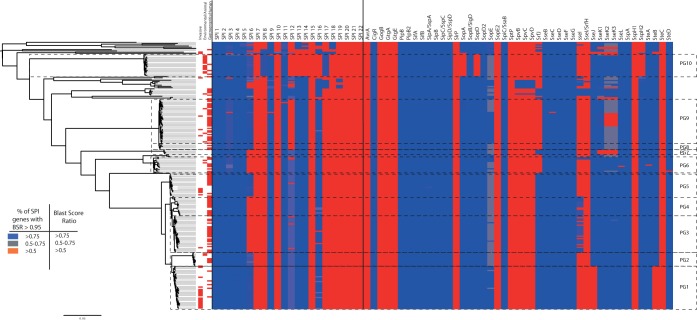
Distribution of SPIs and effectors, with disease phenotypes of isolates indicated immediately to the right of the phylogenetic tree. Color coding for the SPIs is based on the percentage of genes on an SPI that are present in a given isolate (defined as genes with a Blast score ratio to the expected gene of >0.95). Color coding for effectors corresponds to the Blast score ratio recorded for each genome when screened with the reference effectors. Disease phenotypes have been classified into three categories where metadata are available; invasive, gastroenteritis, or environmental. For a full outline of sources, see Table S1 in the supplemental material.

The origin of the confusion around the subdivision of the Paratyphi B complex is not simply that its constituent members possess a shared serotype, it is that a subset of the strains with this serotype has frequently been found to cause invasive disease. Thus, the significance of serotype Paratyphi B is built not only upon the phylogenetic distribution of the *fliC*/*fljB* genes but, also, the distribution of disease phenotypes and virulence genotypes among strains sharing this serotype. To examine the congruence of disease types with phylogenetic positions and known virulence determinants, we examined our sample collection in light of clinical metadata and the known virulence repertoire of these organisms (Fig. 2).

Within PG1, we found that 20/34 of the isolates for which we had clinical metadata were associated with cases of invasive disease. However, while all of the *d*Ta^−^ samples in our data set are found in PG1, it was apparent that there were also cases of invasive disease caused by isolates from outside this PG. Most notably, 5/17 isolates from PG5 (Fig. 2) were also associated with invasive disease. However, performing a pairwise comparison of the rates of invasive disease between PGs using the χ^2^ test reveals that after correcting for multiple sampling, only PG1 shows a significant difference in the rate of invasive disease-causing isolates relative to those of the other PGs (*P* value of 3.9E^−6^ for PG1 versus PG3, 1.2E^−5^ versus PG4, and 0.002 versus PG5 following a Holm-Bonferroni correction). While it is unknown whether the genes affected by the *d*Ta^−^ SNP are causative or merely indicative in terms of the disease phenotype observed, what is clear is that the only Paratyphi B lineage that is strongly associated with invasive disease is PG1. Moving beyond the classical single-site test for subdividing these lineages, we examined the core and pangenomes of the PGs to look for variation in gene contents, specifically in the virulence repertoire of the Paratyphi B complex. We identified the distribution of known *Salmonella* virulence factors associated with invasive disease, including *Salmonella* pathogenicity islands (SPIs) and their associated type three secretion system (TTSS) effector proteins, as well as previously characterized fimbriae (Fig. 2; see also Fig. S4 in the supplemental material).

Our analysis indicates that across all of the PGs, the complement of virulence-related factors is mostly consistent, with all PGs sharing SPI-1 to -5, -9, and -11. All except PG3, -4, and -6 appeared to possess SPI-6, including the type VI secretion system. All of the lineages other than the poultry-associated PG10 possess SPI-12, -13, -14, and -16, while PG10 alone possesses SPI-8 and -17. Across the population, in terms of the SPI complement, there is therefore little to distinguish the apparently more invasive members of PG1 from most of the PGs of the Paratyphi B complex. One possible explanation could be found in the effector and fimbriae proteins.

While the fimbrial content is broadly similar across the sample set (see Fig. S4 in the supplemental material), there is some variation evident when examining TTSS effectors (Fig. 2). Isolates belonging to PG1 to -5 and -7 possess the effector gene *srfJ*, whereas members of other PGs lack it, while PG1 has lost the effectors *sseJ* and *steB*, mirroring losses that have also occurred in *S. enterica* serotype Typhi. This is particularly of note since complementation of *S*. Typhi with a functional *sseJ* decreases cytotoxicity (29), a capability that is thought to aid *S*. Typhi in entering the blood stream. *steB* was recently discovered in *S*. Typhimurium (30), and its role in virulence is yet to be elucidated, but its absence in *S*. Paratyphi B and *S*. Typhi is suggestive of a role that could limit the invasiveness of these lineages were it present. Contrary to previous work (31), we found that *sopE* was not present in all of the isolates of PG1. We did observe that 2 of the members of PG1 had a gene homologous to the *sopE* used in the 2003 paper (31); however, this hit was also found in other isolates across our data set, demonstrating that it is not a suitable marker for identification of invasive strains of Paratyphi B. We also investigated the pangenome to identify any genes that were lost by all of PG1 but present in their close relatives and any genes that have been gained by the PG1 ancestor and retained by all samples. This revealed a limited number of gains and losses within the whole group, with 31 genes being apparently lost across PG1 that are present in every other isolate found with the same serotype. Interestingly, eight of these genes are hydrogenases, and one is in the cellulose biosynthesis pathway (see Table S2)—losses that are also found in other invasive lineages, such as *S*. Typhi and S. Gallinarum (27), as well as in host-adapted strains of other enterobacteriaceae, such as *Yersinia pestis* and *Yersinia enterocolitica* (32). These genes have been previously associated with adaptation to the inflamed mammalian gut (32–34), and so these losses would be consistent with an organism that has adapted or is adapting into an invasive niche.

### Paratyphi B *d*Ta^−^ strains share an ancestor predicted to have existed in the 12th century.

Collectively, the analyses of the virulence-related characteristics of the Paratyphi B PGs revealed a set of lineages that are relatively consistent in terms of their core gene content, with core genomes for PGs with >2 isolates ranging from 3,951 to 4,511 genes. Members of PG1 share 4,236 genes, a level of gene conservation that suggests that the genomes of the Paratyphi B PGs are stable, with a limited amount of gene gain and loss. To place this into a temporal context and to better understand the natural history of the PGs, we used Bayesian Evolutionary Analysis by Sampling Trees (BEAST) to perform a set of population genomic analyses within the PGs where we had sufficient dating information/coverage to produce robust estimates; these were PG1, -2, -5, -8/-9, and -10 (see Fig. S5 in the supplemental material). PG3, -4, and -6 were too diverse to examine using BEAST, and PG7 had too few isolates. These analyses revealed that the invasive lineage, PG1, is more ancient than may have been predicted based upon the low frequency with which its members cause disease today. The median date predicted by BEAST (35) for the most recent common ancestor (MRCA) of this group is 1188 AD (95% confidence interval [CI], 1799 AD to 469 BC), implying that PG1 is older than the Paratyphi A serotype, whose common ancestor is dated to ~450 years ago (36). This is an interesting finding for two reasons. First, this suggests that the core genome of 4,236 genes has been conserved within PG1 for over 750 years, implying that the genome is very stable. Second, this finding is notable given that Paratyphi A strains are now more frequently isolated than Paratyphi B strains but Paratyphi B appears to have emerged first. In comparison, the other main PGs of clinical significance appear to have emerged more recently. PG8/-9 have an MRCA in 1726 (95% CI, 1880 to 1448), while the poultry-associated PG10 has an MRCA that dates to 1977 (95% CI, 2001 to 1859), pointing toward its recent emergence as a pathogen associated with intensive farming of poultry. Finally, the most recent strains in PG5 have an MRCA dated to the beginning of the 1980s (95% CI, 2008 to 1738). This observation suggests that the clone has recently expanded, a surprising observation given the lack of antimicrobial resistance found in this group. This observation is true of most of the PGs examined. We see generally low levels of inter-PG recombination (see Fig. S6) and very limited evidence of the acquisition of antimicrobial resistance (see Fig. S7). Our analysis reveals that the acquisitions of resistance elements have been single, local events that occurred within PG3 (SGI-1) and PG10 (a chromosomal class 2 integron along with extended-spectrum β-lactamase [ESBL]-encoding plasmids). Subsequently, we only observe evidence for vertical inheritance, with no evidence of the spread of these elements to other lineages.

### Conclusion.

Using next-generation sequencing, we have been able to uncover much of the genomic basis for the confusion around this serotype. It is clear, when examining the data on a genomic scale, that strains possessing a serotype with the antigenic formula 1,4,[5],12:b:[1,2] can be subdivided into at least 10 groups. Unlike MLST, which could only ever indicate that these groupings were diverse, whole-genome sequencing provides us with the capacity to quantify the divergence between groups and place the resulting data in the context of other inter- and intra-*Salmonella* serotype variation. The analysis presented here demonstrates the advantages of using whole-genome approaches over eBURST groups (eBGs) for subdividing *S*. Paratyphi B. eBGs have previously been suggested as a basis for diagnostics for *S. enterica* (7), but the results presented here clearly show that eBGs do not distinguish *d*Ta^−^ strains from *d*Ta^+^ strains, making these unsuitable for rapid diagnostics from either traditional MLST or genome sequence data. This analysis also reveals clearly that, over the last 1,000 years, there have been a set of independent clonal expansions of lineages that share the common serotype with the formula 1,4,5,12:b:1,2, and based upon an examination of the flagellum genes responsible for the phase 1 and phase 2 components of the serotype, these expansions have been predated by recombination events importing one or other of these genes into different chromosomal backgrounds. The number of lineages carrying serotype Paratyphi B is suggestive of the fact that this serotype is successful in a number of niches. Within the population of strains carrying serotype Paratyphi B, there are strains that have been isolated from humans, where they caused invasive disease or gastroenteritis, from poultry, and from aquatic organisms and/or the aquatic environment. This large number of lineages carrying the same serotype is suggestive that serotype switching is more frequent than in other successful lineages, such as *S*. Typhimurium, which have remained discrete lineages.

Conversely, we find that both the core genome more generally and lipopolysaccharide (LPS) genes specifically are remarkably stable, with the genes within the O-antigen cluster producing a phylogeny mirroring that produced by the core genome. Of the 10 PGs that we identified, one (PG1) comprised the canonical Paratyphi B group. This group is relatively closely related to PG2 to -5, producing a larger cluster of lineages where the intergroup divergence is comparable to that found in other closely related host generalist/host-specialized lineages, such as the *S*. Enteritidis/Gallinarum group. Examining phylogenetic clustering in the context of disease type, it is clear, however, that invasive disease is not limited to PG1 to -3; PG5, -6, -9, and -10 can all cause invasive disease, albeit to various extents. However, based upon our sample data set, only PG1 is significantly associated with invasive disease. While isolates that are *d*Ta^−^ have historically been classified as “invasive,” isolates from PG5 may represent a lineage, similar to the invasive sequence type 313 (ST313) of *S*. Typhimurium (37), that has a higher propensity to cause invasive disease given particular host-associated factors (advanced age, suppressed immune system, etc.) but which is not an “invasive” lineage *per se*. It is our hope that by defining the population structure of this serotype, it will be possible to investigate this question more precisely in the future.

Although there are fewer clues as to the genomic basis for the difference in disease type than may be expected, given the difference in symptoms and infection sites, there are several meaningful signals—most notably, the variations in the complements of effectors and the presence of SPIs—that point toward the genomic differences that underpin the observed disease types. The variations in SPI and effector presence in particular are significant for two reasons. First, the degradation of these elements in lineages may be mechanistic, and since they are in virulence-related factors, this degradation may relate directly to the invasiveness of the isolates. Second, because SPIs/effectors are differentially present within lineages within the Paratyphi B complex, they may provide possible diagnostic testing targets that are linked to the machinery actually used to cause disease, rather than characteristics which are probably related to disease but not causal, such as the tartrate test. In particular, the presence/absence of effectors *sseJ* and *steB* would appear to provide a simple mechanism for identifying PG1 (and *S*. Paratyphi A and *S*. Typhi) using PCR, potentially reducing the time taken to detect BSL3 *d*Ta^−^ *S*. Paratyphi B from 13 days to a few hours in virtually any laboratory in the world.

This work underlines the difficulty posed when genomic approaches are not used to subdivide lineages and exemplifies the challenges that face classical typing, reinforcing the need for unambiguous molecular methods for characterizing members of the Paratyphi B complex. This work also demonstrates both the limitations of genomics alone to unpick the complex biological processes that translate genotype to phenotype and a methodological framework that can be used to explore other polyphyletic *S. enterica* serotypes where variations in gene content have been observed, such as 4,[5]12:b:−, using PCR-based approaches (38). The lack of consistent genomic differences between PG1 to -5 suggests that the invasiveness of PG1 may be due to hitherto undiscovered virulence determinants or to other factors, such as transcriptional control. The loss of hydrogenases and elements of the cellulose biosynthesis pathway—established components of the blueprint for invasive salmonellae—is also suggestive that metabolic changes have also occurred within PG1 as these organisms adapt to an invasive niche but that Paratyphi B may represent a sort of evolutionary halfway house, sitting somewhere between a host-adapted and invasive serotype. This work therefore provides a basis for reinterpreting what we already know about invasive salmonellae, providing a simple practical basis for distinguishing the invasive PG1 strains from other, noninvasive strains, while building a foundation for future work to better understand what makes *d*Ta^−^/PG1 strains so invasive compared to their close *d*Ta^+^ relatives in PG2 to -5.

## MATERIALS AND METHODS

### Samples.

In order to explore the population structure of *Salmonella* Paratyphi B, as defined by isolates sharing the antigenic formula 1,4,[5],12:b:[1,2], we sequenced the genomes of a collection of 191 isolates collected from the United Kingdom, France, Spain, Ireland, and Canada that encompasses all currently known MLST eBURST groups that are labeled as being serotype Paratyphi B, including both monophasic and diphasic strains. In addition to the deliberate selection of a range of historical strains that covers the full population of this serotype (*n* = 81), we also collected samples from clinical episodes of disease in the United Kingdom (*n* = 63) and Spain (*n* = 33), as well as isolates from poultry-associated disease reported previously (*n* = 14) (see Table S1 in the supplemental material for a full list of the sources and strains used in this study) (11). All of our strains were classically serotyped prior to sequencing by the respective originating reference laboratory: the Spanish National Reference Laboratory, the Institut Pasteur, the *Salmonella* Reference Laboratory of Health Protection England, or the National Microbiology Laboratory, Public Health Agency of Canada. This classical serotyping was confirmed using genome sequencing. To provide a wider phylogenetic context, we also include within our study a further 27 *Salmonella enterica* isolates, representing isolates carrying 18 different serotypes. Of these, 21 are previously published strains and 6 represent new sequences that were generated as part of this study. Additionally, we include a further 2 isolates that are of serotype Dublin/Enteritidis but are grouped by MLST with the predominantly Paratyphi B eBG 32 (see Table S1 for a full list of strains, with accession numbers, serotype information, and other relevant metadata).

### Genome sequencing.

The genomes were sequenced using the Illumina sequencing platform, with Genome Analyzer IIx (GAIIx), HiSeq, and MiSeq instruments being used to sequence isolates to approximately 200× coverage, as described previously (39). The samples were generated with a mean insert size of between 200 and 300 bp, and depending upon the instrument used, underwent 2 × 50 bp paired-end sequencing (GAIIx), 2 × 100 bp paired-end sequencing (HiSeq), or 2 × 250 bp paired-end sequencing (MiSeq). The data were assembled *de novo* using Velvet (40), with assemblies improved using the Velvet Columbus module and the software package iCORN (41).

### Phylogenetic analysis and variant calling.

Using the tool Snippy running on the Cloud Infrastructure for Microbial Bioinformatics (46), we performed mapping against the reference Paratyphi B strain SPB7, identifying 147,963 positions that are present in all samples in our collection but vary in at least one isolate. Extracting these variable sites, we removed putative regions of recombination, using the tool Gubbins (25), to produce a set of core positions that are free from recombination. These were then used to generate a phylogenetic tree using RAxML, which was generated using a general time-reversible model with gamma correction (GTR-gamma) for between-site heterogeneity. Additionally, in order to better quantify the core/accessory genomes for the isolate collection, we made use of the large-scale Blast score ratio (LS-BSR) tool (42), which was run against the assembled genomes of the complete data set. The matrix generated by LS-BSR was then processed using the PanGP tool to visualize the size of the core genome across the data set.

### Comparative genomics.

We identified genes of interest through literature searches and examination of annotated *Salmonella* genomes, producing a set of query sequences to explore the SPI complement and to allow us to find and extract fimbriae and effectors from our *de novo* assemblies. To examine the SPI contents, we identified the genes present within the pangenome that are carried on SPI-1 to -22, and using these, calculated the number of genes associated with each SPI that were represented in each sample. To examine fimbria, effector, and flagellum genes and the O-antigen gene cluster, we located and screened genes or regions of interest using the LS-BSR tool (42). We then visualized the results using a simple script that converts comma-separated-value (CSV) tables into vector graphics, developed in house (fimbriae, SPIs, and effectors—available at https://github.com/tomrconnor/Basicscripts). To extract genes, we performed a Blast search across the assembled genomes for targets of interest using an approach described previously (32), extracting sequences, aligning them with MUSCLE (43), and generating trees using a GTR-gamma model under PhyML (44). To confirm the presence of *sopE* in Paratyphi B samples, we used SRST2 in addition to the approach described above. SRST2 (45) uses a mapping-based approach to map sequence reads against a set of target sequences, identifying whether there is evidence for those sequences within a file of sequence reads from a genomic sample. This complements the assembly-based approach and compensates for potential limitations around the detection of genes from assemblies. The results generated from this analysis are reproduced in Table S3 in the supplemental material.

### Population genetic analyses.

To subdivide the population, we made use of the software package Bayesian Analysis of Population Structure (BAPS) (26). We provided the software with the mapping-based SNP alignment for the data set prior to the removal of recombinations and performed a hierarchical BAPS (26) run to 2 levels with a maximum number of 50 populations, using the second level of clustering to define phylogroups (PGs) from the population. We imposed an artificial limitation that a phylogroup must contain a minimum of 2 isolates. The analysis was run three times to confirm the clustering results. As well as identifying a set of PGs containing 2 isolates or more, the algorithm also identified a number of other isolates that may or may not constitute new PGs, and we anticipate that these candidate PGs will be confirmed (or not) over time. To estimate a dated phylogeny, we made use of BEAST (35) 1.8 and performed the analysis on an SNP alignment for the isolates that we had good dating information on. Performing BEAST on each PG individually, we used three chains with a total length of 100,000,000 states each and with trees sampled every 10,000 states for each data set. To identify the best combination of models to use, for each sample set, we performed this analysis for constant and lognormal clock models and for constant, logistics, expansion, exponential, and skyline demographic models. For each data set, the runs that converged and generated effective sample size (ESS) values of >200 were compared, and the best model was selected based on the AICM (Akaike’s information criterion through Markov chain Monte Carlo), calculated using Tracer. The best models determined on this basis for each run were as follows: a lognormal clock and skyline model for PG1, a lognormal clock and expansion model for PG2, a lognormal and skyline model for PG5, a lognormal and skyline model for PG8/-9, and a lognormal and expansion model for PG10. In the case of PG3 and -4, BEAST did produce results, but the predicted MRCA for the best model was over 300 years in the past, despite the fact that we only had samples going back 15 to 20 years. In both of these cases, we concluded that our sample was too diverse to derive accurate BEAST results. In the case of PG6, we did not have enough samples with good date information for BEAST to produce usable results. For the final selected BEAST runs, the ESS values were >>200 in all cases. The tree files were combined using LogCombiner, processed using TreeAnnotator, and visualized with FigTree, all tools that are part of or published with the BEAST package and are freely available from http://beast.bio.ed.ac.uk.

### Accession numbers.

The sequence data for this project have been deposited in the European Nucleotide Archive. Please see Table S1 for accession numbers and metadata for the individual strains examined.

## SUPPLEMENTAL MATERIAL

Figure S1  Pan- and core genome graphs showing the rarefaction curves for the core (green) and accessory (blue) genomes of the data set. Computed using LS-BSR and visualized by PanGP. Download Figure S1, PDF file, 0.2 MB

Figure S2  Phylogeny showing the SNP counts per branch, based on ancestral reconstruction using ACCTRAN. Download Figure S2, PDF file, 0.03 MB

Figure S3  Maximum-likelihood phylogeny of genes located around *fliC* and *fljB* in Paratyphi B, from a concatenated alignment of the genes produced using MUSCLE. The tree was drawn using PhyML, with a GTR-gamma model of between-site variation. Download Figure S3, PDF file, 0.05 MB

Figure S4  Distribution of genes forming different (indicated) fimbriae across the tree, next to the maximum-likelihood tree generated for the data set, as described in the legend to Fig. 1. It is important to note that there are two genes named *steB* in *Salmonella enterica*; the *ste* fimbriae indicated on this figure include a gene named *steB* that encodes an 899-amino-acid outer membrane usher protein; this gene is different than the 133-amino-acid secreted effector protein-encoding *steB*, found in *S*. Typhimurium but not in isolates belonging to PG1 in this study (*S*. Typhi or *S*. Paratyphi A). Download Figure S4, PDF file, 0.1 MB

Figure S5  Dated phylogenies charting the emergence of PGs, showing the maximum clade credibility tree predicted by BEAST for the six clusters of strains within our data set: PG1 (a), PG2 (b), PG5 (c), PG8/-9 (d), and PG10 (e). PG8/-9 were combined due to their relatively close phylogenetic relationship and the limited number of samples in PG9 which would have precluded an individual BEAST analysis on this cluster. Download Figure S5, PDF file, 0.2 MB

Figure S6  Output from BRAT NextGen showing the limited amounts of population-wide signals for recombination across the data set. Download Figure S6, PDF file, 0.9 MB

Figure S7  Blast score ratios obtained when the data set was screened against a panel of antimicrobial resistance (AMR) genes. These are placed into context next to the maximum-likelihood tree generated for the data set, as described in the legend to Fig. 1. Download Figure S7, PDF file, 0.1 MB

Table S1  Complete list of strains used in this study, with metadata and accession numbers. Table S1, XLSX file, 0.1 MB

Table S2  a list of the genes identified as absent in isolates belonging to PG1 but present in isolates belonging to PG2 to -10. Table S2, XLSX file, 0.02 MB

Table S3  SRST2 results showing *sopE* hits from Paratyphi B genomes. Table S3, XLSX file, 0.04 MB
