# Supplementary figures and images for: What’s in a Name? Species-Wide Whole-Genome Sequencing Resolves Invasive and Noninvasive Lineages of Salmonella enterica Serotype Paratyphi B
Source: mBio. 2016 Aug 23;7(4):e00527-16. doi: 10.1128/mBio.00527-16 (PMC4999539; doi:10.1128/mBio.00527-16)

Figure S1

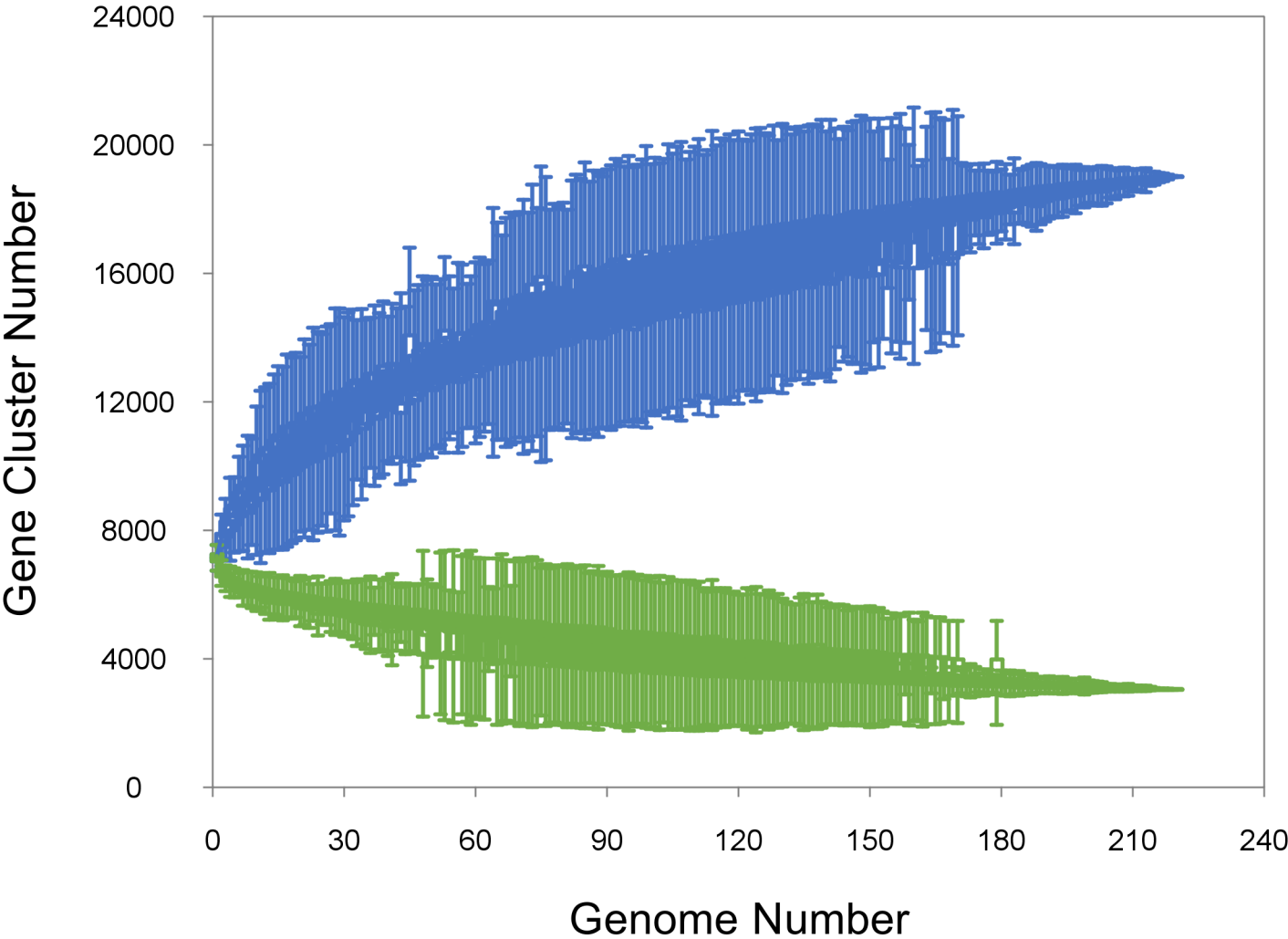

Supplement: Figure S1 — Pan- and core genome graphs showing the rarefaction curves for the core (green) and accessory (blue) genomes of the data set. Computed using LS-BSR and visualized by PanGP. Download [file mbo004162949sf1.pdf]

7000 0

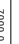

|      | PG1   | PG2   | PG3   | PG4   | PG5   | PG6   | PG7   | PG8   | PG9   | PG10 |
|------|-------|-------|-------|-------|-------|-------|-------|-------|-------|------|
| PG1  | x     |       |       |       |       |       |       |       |       |      |
| PG2  | 5320  | x     |       |       |       |       |       |       |       |      |
| PG3  | 4399  | 8733  | x     |       |       |       |       |       |       |      |
| PG4  | 3902  | 8236  | 1017  | x     |       |       |       |       |       |      |
| PG5  | 5628  | 9962  | 5709  | 4692  | x     |       |       |       |       |      |
| PG6  | 28232 | 32566 | 28313 | 27296 | 26700 | x     |       |       |       |      |
| PG7  | 39002 | 43336 | 39083 | 38066 | 37470 | 27670 | x     |       |       |      |
| PG8  | 48654 | 52988 | 48735 | 47718 | 47122 | 37322 | 35308 | x     |       |      |
| PG9  | 48608 | 52942 | 48689 | 47672 | 47076 | 37276 | 35262 | 30973 | x     |      |
| PG10 | 67393 | 71727 | 67474 | 66457 | 65861 | 56061 | 54047 | 49987 | 49941 | x    |

Supplement: Figure S2 — Phylogeny showing the SNP counts per branch, based on ancestral reconstruction using ACCTRAN. Download [file mbo004162949sf2.pdf]

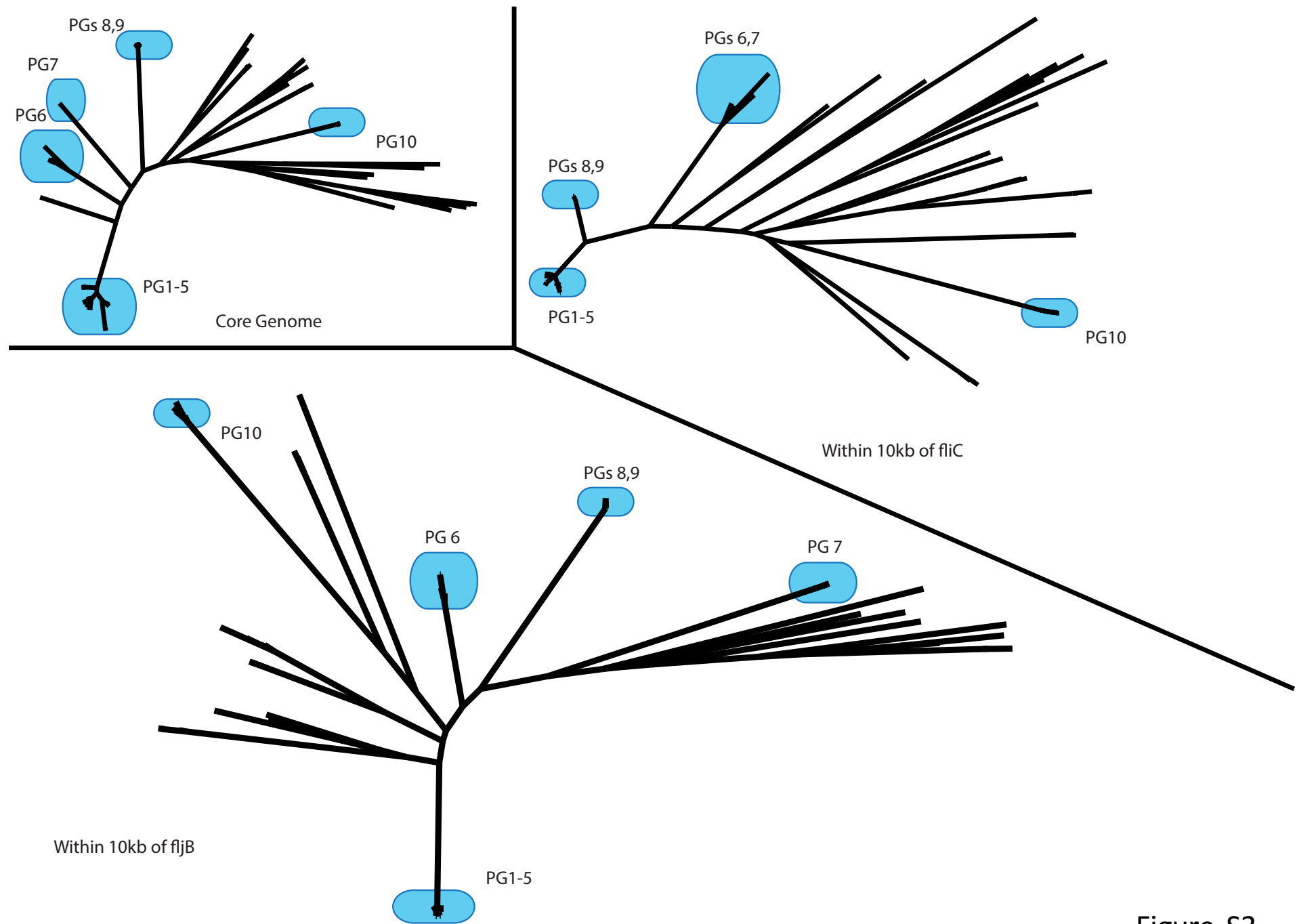

Figure S3

Supplement: Figure S3 — Maximum-likelihood phylogeny of genes located around fliC and fljB in Paratyphi B, from a concatenated alignment of the genes produced using MUSCLE. The tree was drawn using PhyML, with a GTR-gamma model of between-site variation. Download [file mbo004162949sf3.pdf]

Figure S4

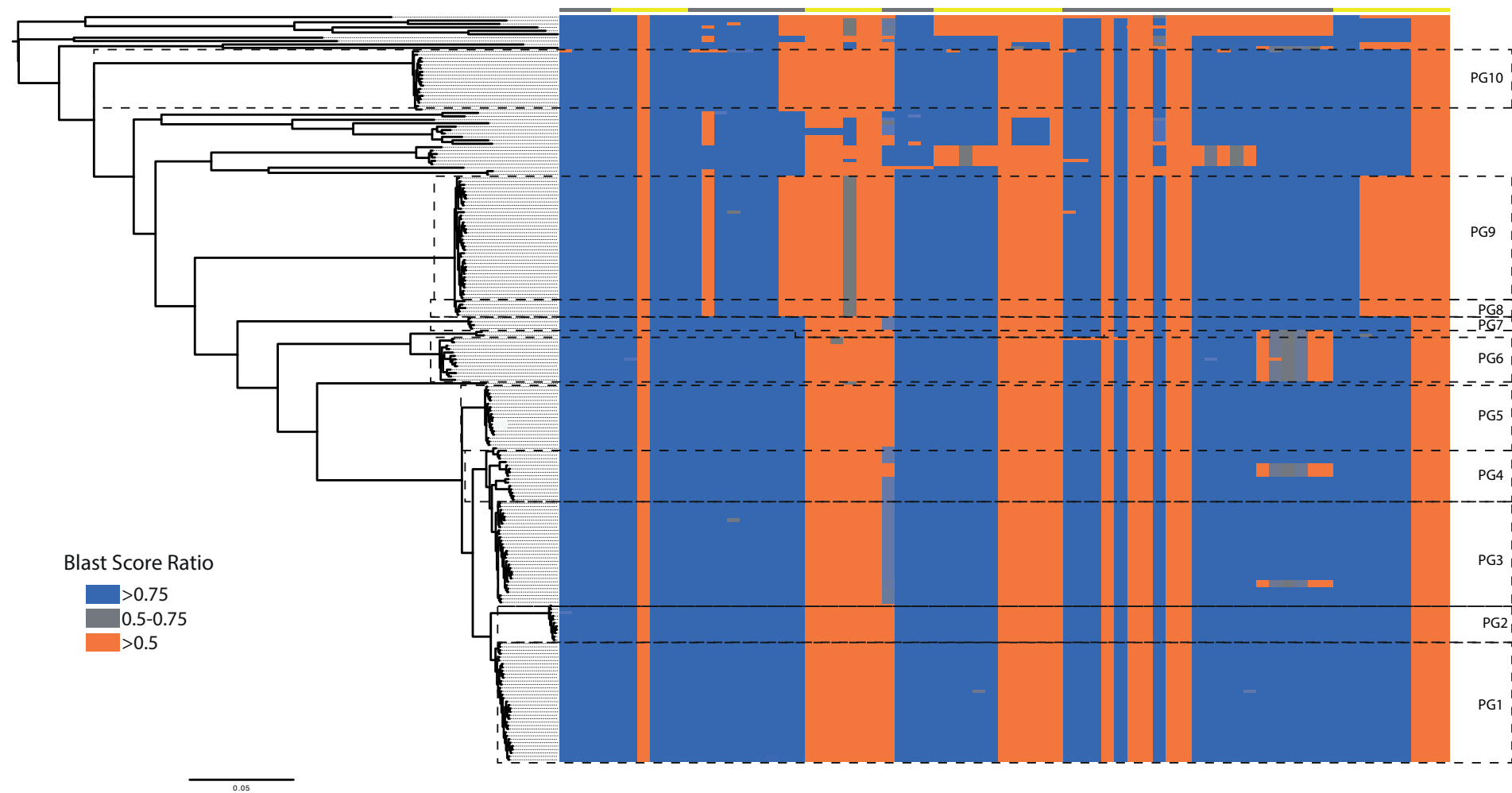

Supplement: Figure S4 — Distribution of genes forming different (indicated) fimbriae across the tree, next to the maximum-likelihood tree generated for the data set, as described in the legend to Fig. 1. It is important to note that there are two genes named steB in Salmonella enterica; the ste fimbriae indicated on this figure include a gene named steB that encodes an 899-amino-acid outer membrane usher protein; this gene is different than the 133-amino-acid secreted effector protein-encoding steB, found in S. Typhimurium but not in isolates belonging to PG1 in this study (S. Typhi or S. Paratyphi A). Download [file mbo004162949sf4.pdf]

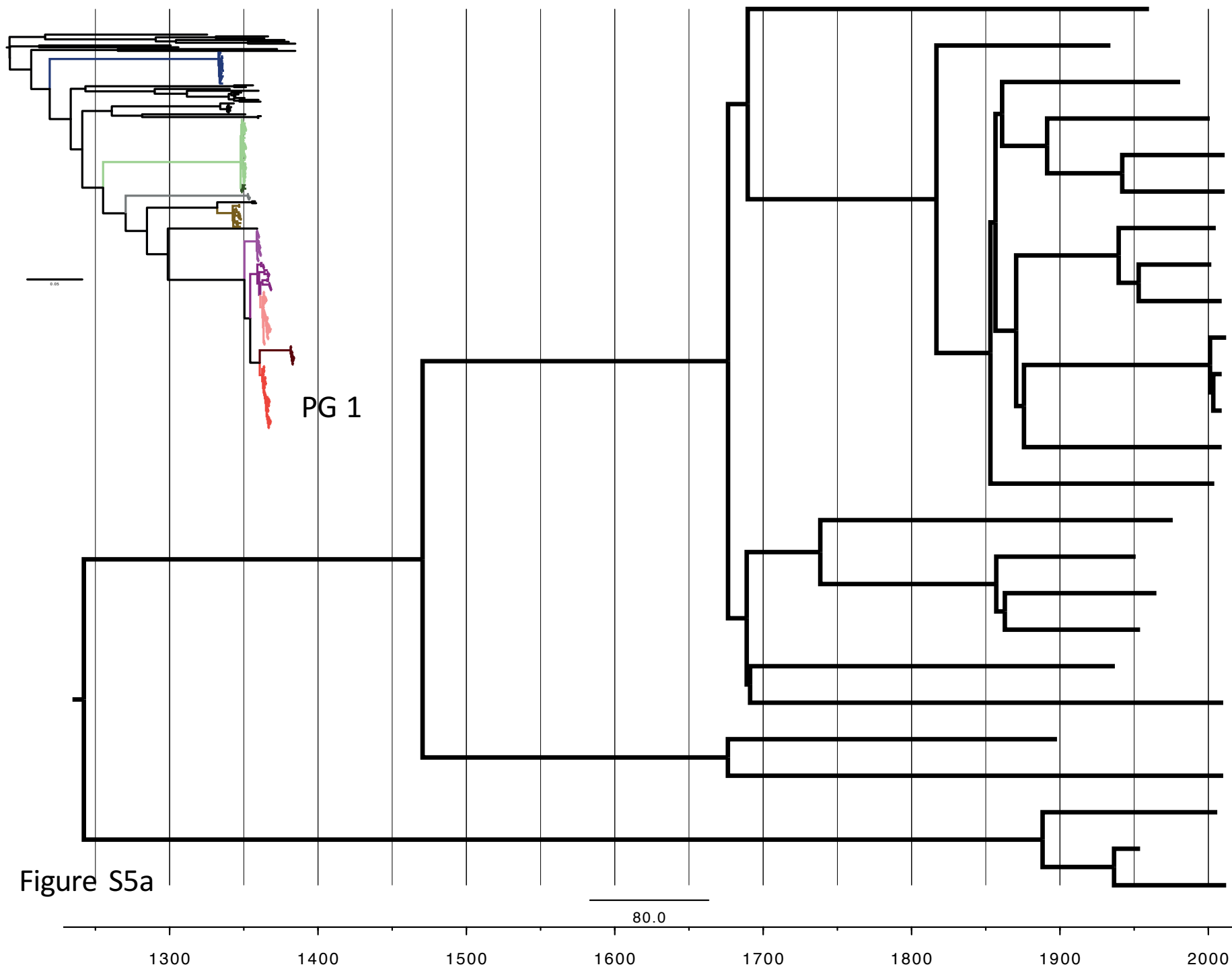

Figure S5b

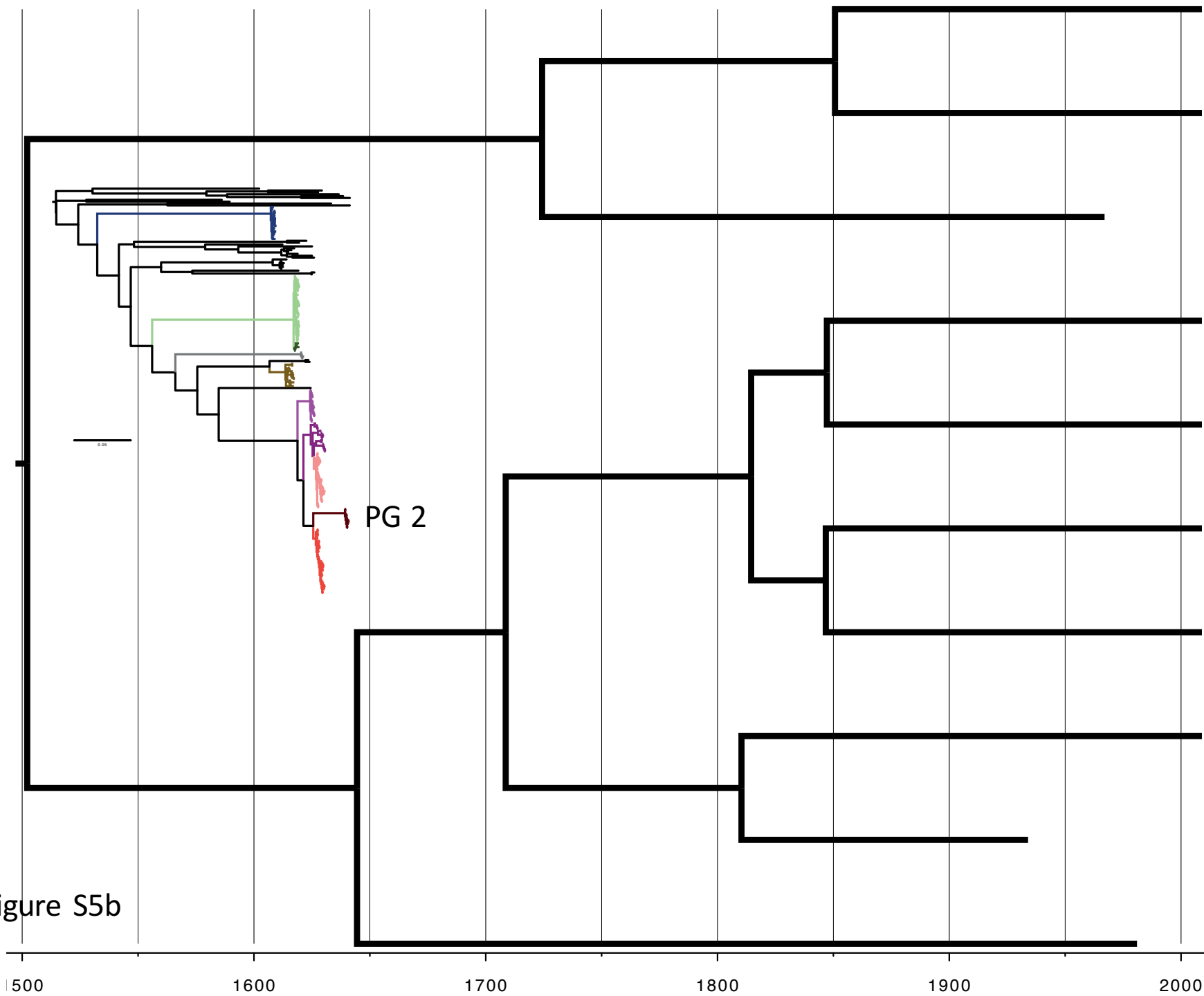

Figure S5c

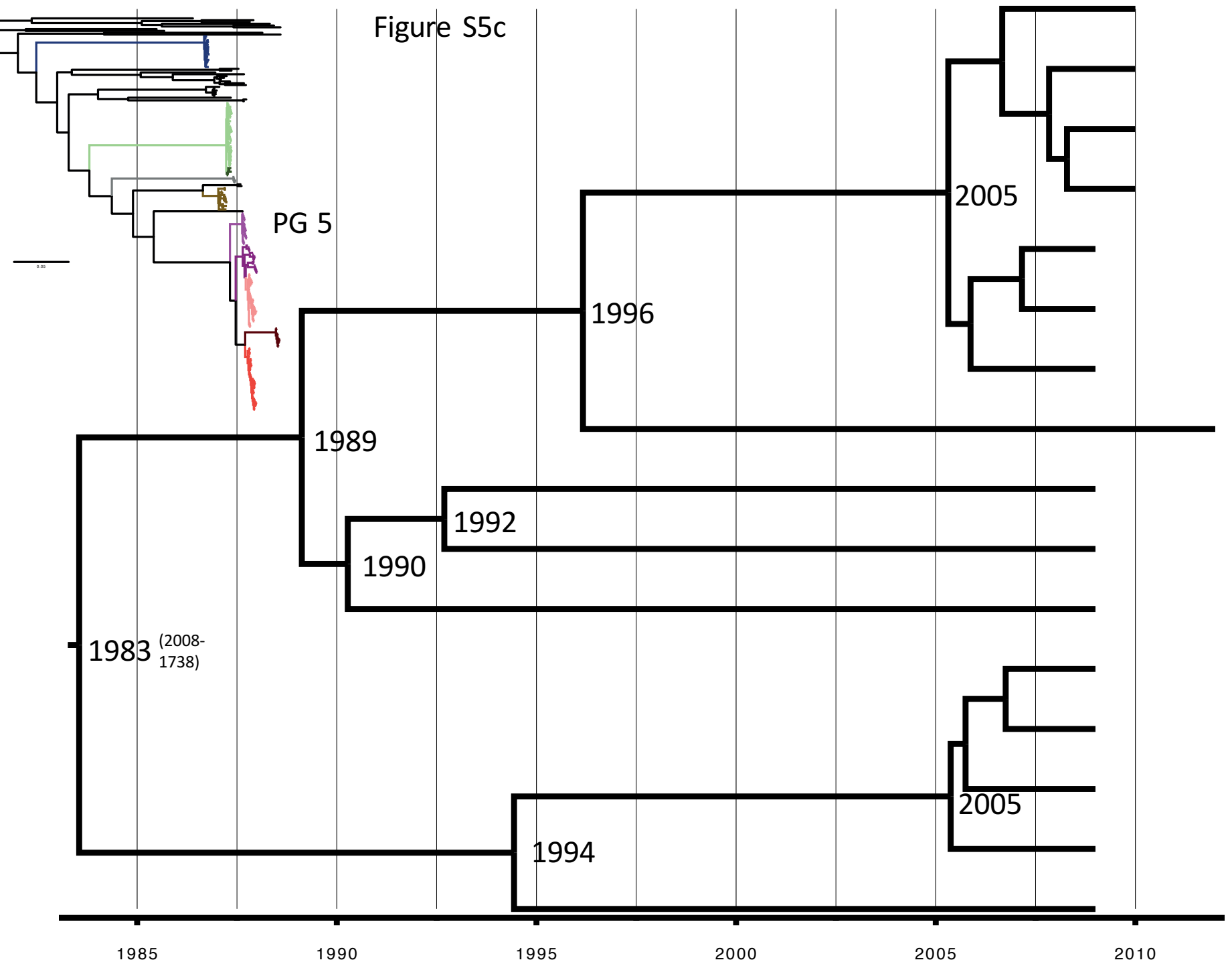

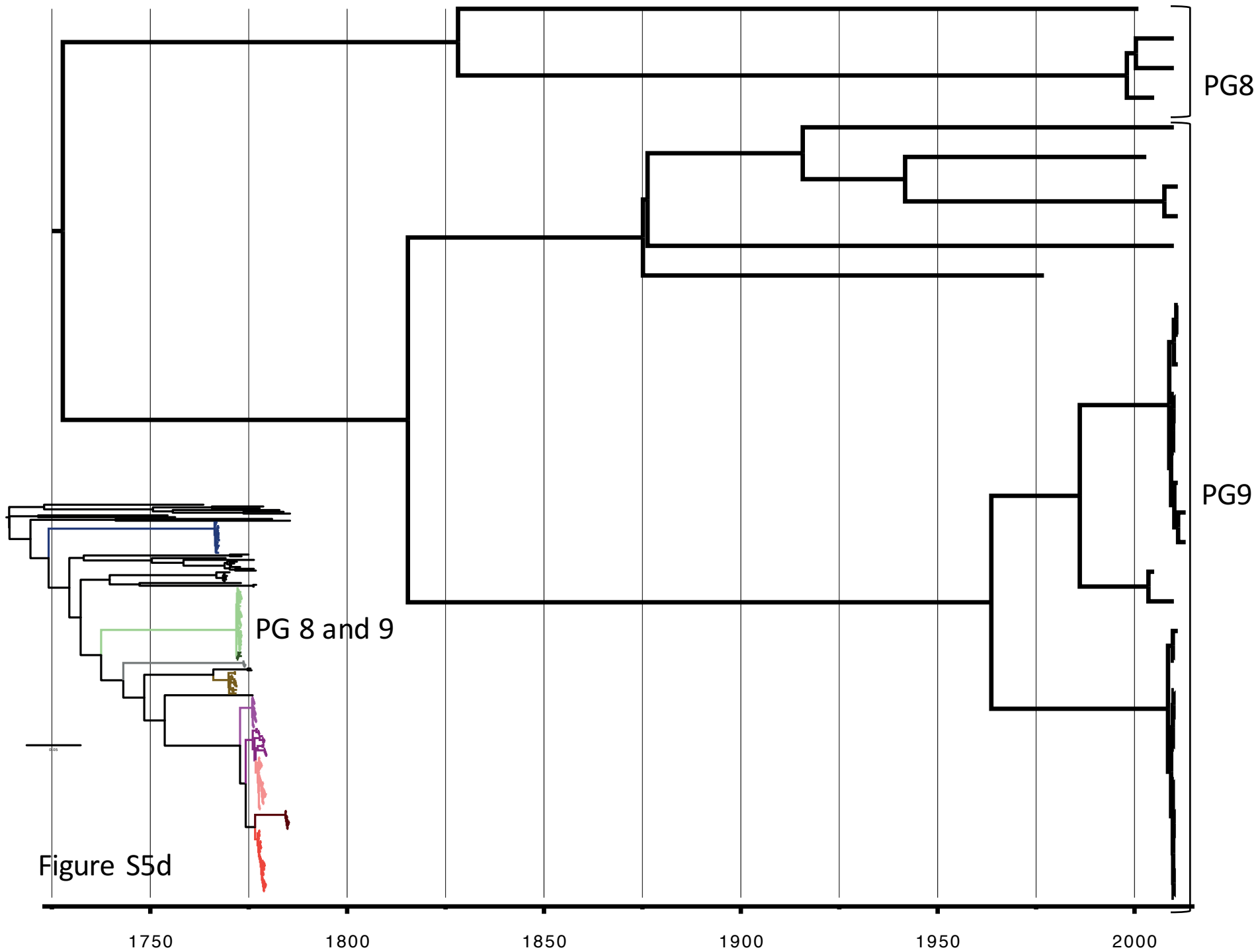

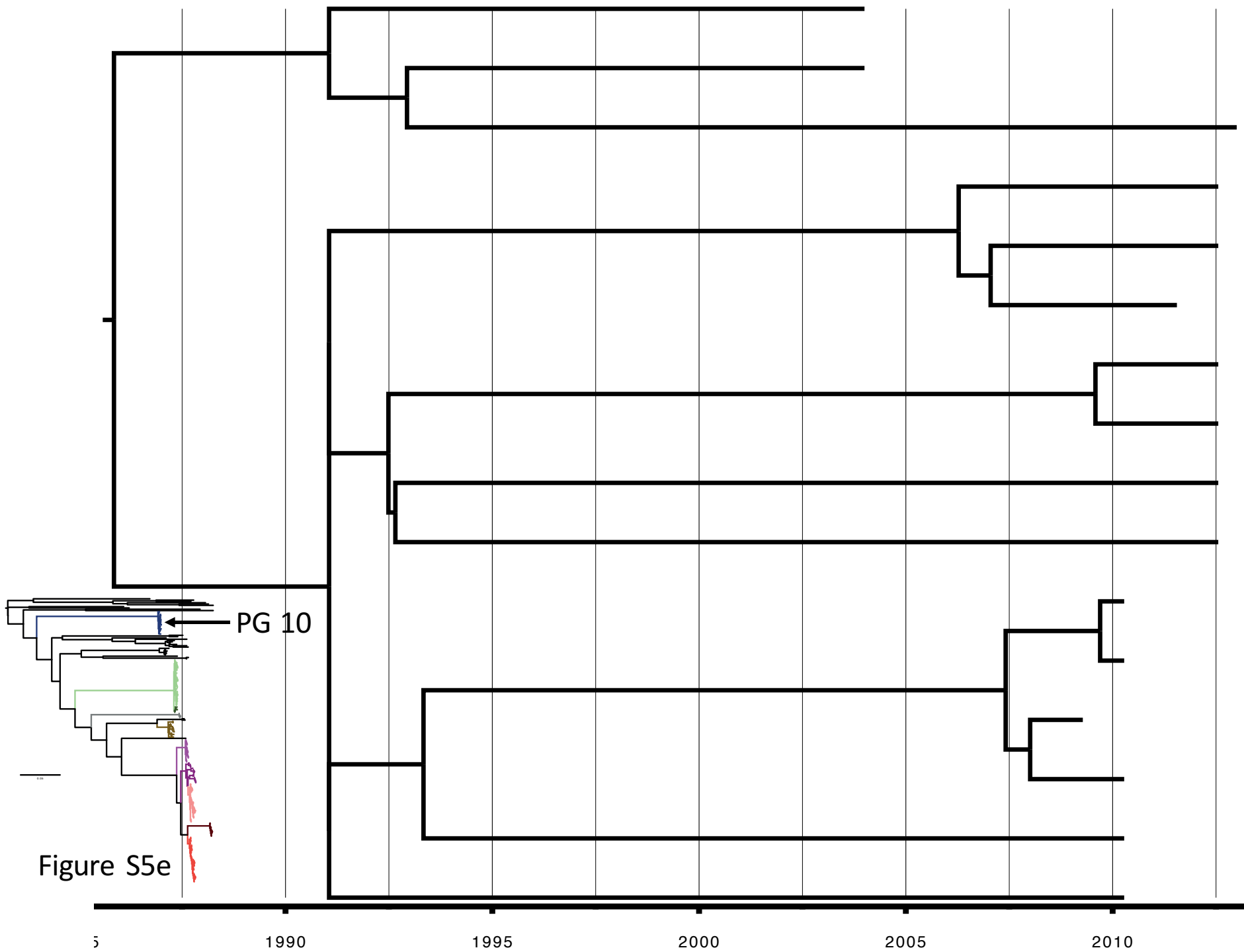

Supplement: Figure S5 — Dated phylogenies charting the emergence of PGs, showing the maximum clade credibility tree predicted by BEAST for the six clusters of strains within our data set: PG1 (a), PG2 (b), PG5 (c), PG8/-9 (d), and PG10 (e). PG8/-9 were combined due to their relatively close phylogenetic relationship and the limited number of samples in PG9 which would have precluded an individual BEAST analysis on this cluster. Download [file mbo004162949sf5.pdf]

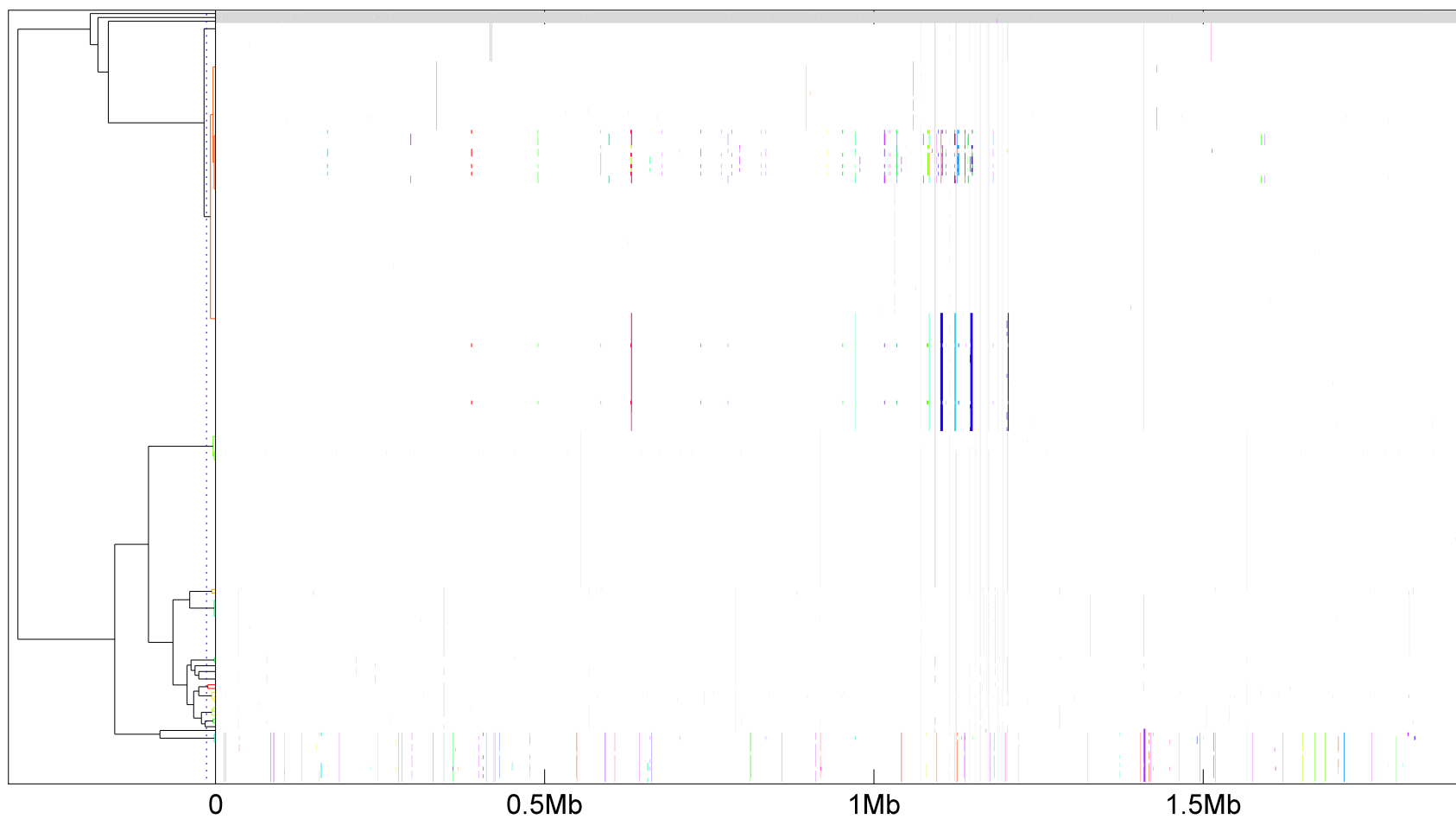

Figure S6

Supplement: Figure S6 — Output from BRAT NextGen showing the limited amounts of population-wide signals for recombination across the data set. Download [file mbo004162949sf6.pdf]

## Figure S7

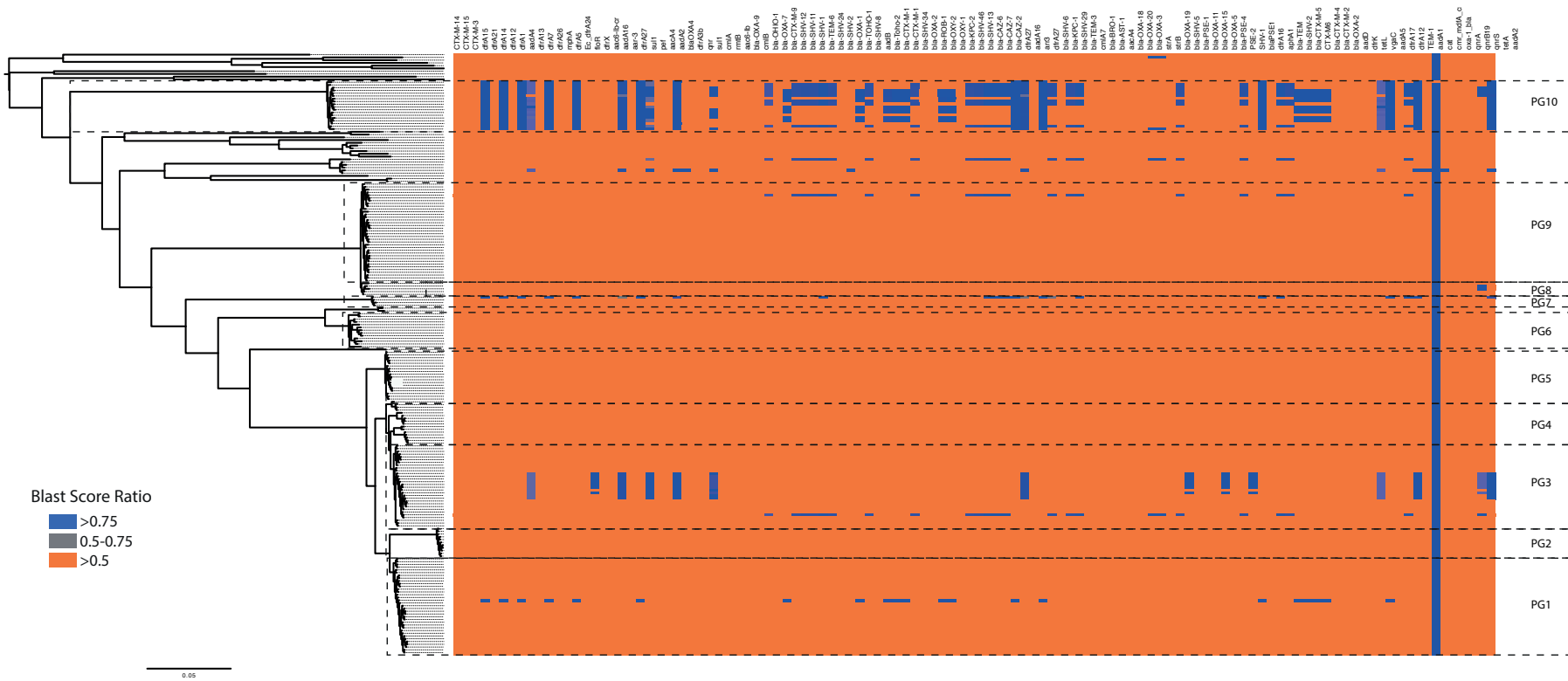

Supplement: Figure S7 — Blast score ratios obtained when the data set was screened against a panel of antimicrobial resistance (AMR) genes. These are placed into context next to the maximum-likelihood tree generated for the data set, as described in the legend to Fig. 1. Download [file mbo004162949sf7.pdf]
